# Supplementary material for: Assessment of Electrical Brain Activity of Healthy Volunteers Exposed to 3.5 GHz of 5G Signals within Environmental Levels: A Controlled–Randomised Study
Source: Int J Environ Res Public Health. 2023 Sep 21;20(18):6793. doi: 10.3390/ijerph20186793 (PMC10530694; doi:10.3390/ijerph20186793)
Supplement: Supplementary file 1 [file ijerph-20-06793-s001.zip › ijerph-2557264-SI.pdf]

Supplementary Materials

Table S1. Volunteers' characteristics.

Subjects n°	Code	Sex (F/M)	Age (years)	Weight (Kg)	Height (cm)	Body Mass Index (kg/m^2)
**1**	sub-S01	M	22	70	178	22.09
**2**	sub-S02	M	29	56	170	19.38
**3**	sub-S03	M	23	84	167	30.12
**4**	sub-S04	M	31	75	170	25.95
**5**	sub-S05	M	26	70	178	22.09
**6**	sub-S06	F	25	65	165	23.88
**7**	sub-S07	M	30	89	173	29.74
**8**	sub-S08	M	29	63	183	18.81
**9**	sub-S09	M	34	95	193	25.50
**10**	sub-S10	F	25	67	178	21.15
**11**	sub-S11	M	35	88	187	25.17
**12**	sub-S12	M	18	72	185	21.04
**13**	sub-S13	M	25	70	172	23.66
**14**	sub-S14	F	21	55	171	18.81
**15**	sub-S15	M	30	64	183	19.11
**16**	sub-S16	M	18	77	186	22.26
**17**	sub-S17	F	25	50	157	20.28
**18**	sub-S18	M	34	88	168	31.18
**19**	sub-S19	M	31	65	175	21.22
**20**	sub-S20	M	30	83	185	24.25
**21**	sub-S21	F	34	56	159	22.15
**22**	sub-S22	F	23	61	159	24.13
**23**	sub-S23	M	20	64	174	21.14
**24**	sub-S24	F	26	54	165	19.83
**25**	sub-S25	F	28	65	163	24.46
**26**	sub-S26	F	32	50	164	18.59
**27**	sub-S27	F	22	52	170	17.99
**28**	sub-S28	F	31	53	169	18.56
**29**	sub-S29	F	25	53	167	19.00
**30**	sub-S30	F	26	65	160	25.39
**31**	sub-S31	F	21	95	166	34.48
**32**	sub-S32	F	24	58	148	26.48
**33**	sub-S33	F	24	60	157	24.34
**34**	sub-S34	F	27	80	168	28.34
**Mean**			**26.59**	**68.00**	**170.97**	**23.25**
**Standard deviation**			**4.67**	**13.25**	**10.18**	**4.06**
**Standard error of mean**			**0.80**	**2.27**	**1.75**	**0.70**
